# Annual PM_2.5_ exposure and clinical, laboratory, and stroke-risk outcomes in pediatric sickle cell disease

**DOI:** 10.1172/jci.insight.190648

**Published:** 2025-06-09

**Authors:** Paul E. George, Grace Kalmus, Joseph Lipscomb, David H. Howard, Benjamin Kopp, Wilbur A. Lam, Stefanie Ebelt

**Affiliations:** 1 Emory University, Rollins School of Public Health, Atlanta, Georgia, USA.; 2 Aflac Cancer and Blood Disorders Center, Children’s Healthcare of Atlanta (CHOA), Georgia, USA.; 3 Emory University, School of Medicine, Atlanta, Georgia, USA.

**Keywords:** Clinical Research, Hematology, Epidemiology

## Abstract

Sickle cell disease (SCD) causes severe morbidity and early mortality, yet it varies phenotypically. Both air pollution and SCD affect the cardiorespiratory, inflammatory, and endothelial systems; however, limited evidence exists on the effect of long-term air pollution exposure in SCD. We hypothesized that annual ambient (outdoor) concentrations of fine particulate matter (PM_2.5_), particles with a diameter of 2.5 μm or less, at a child’s home would be significantly associated with worse clinical, laboratory, and stroke-risk imaging outcomes. Patient data for this retrospective study were obtained from a cohort of children with SCD (from 2010 to 2019). Annual PM_2.5_ exposure was estimated using remote-sensing air pollution datasets. Statistical analyses employed fixed effects multivariable models, offering a robust approach to isolate the effect of PM_2.5_ exposure. The final cohort included 1,089 children with SCD. Higher annual PM_2.5_ concentrations were significantly associated with more annual hospital days, higher likelihood of hospitalization and abnormal stroke-risk screening, and elevated inflammatory markers. Of note, hydroxyurea use mitigated the inflammatory response to PM_2.5_ but did not mitigate the effect of PM_2.5_ on clinical outcomes. Importantly, the elevated stroke risk associated with PM_2.5_ exposure persisted, even among children receiving hydroxyurea therapy, highlighting a critical concern in pediatric SCD management. These results underscore the clinical importance of addressing environmental factors for comprehensive SCD care.

## Abstract 2

Children with sickle cell disease exposed to higher air pollution (PM₂.₅) experience more hospitalizations and increased stroke risk, highlighting environmental health impacts.

## Introduction

Sickle cell disease (SCD) is one of the commonest genetic disorders, with approximately 100,000 Americans and an estimated 7 million people worldwide living with the disease (1). As a monogenic disorder, SCD arises from a mutation in the *HBB* gene that encodes hemoglobin. Despite being a monogenic defect, SCD is phenotypically variable (2). While nearly everyone with SCD experiences ongoing morbidity and reduced life expectancy, the severity of the disease, especially in childhood and adolescence, is quite variable. Some children and young adults experience frequent pain crises, severe lung injury (acute chest syndrome), frequent hospitalizations, and debilitating strokes, whereas others are rarely hospitalized and are less affected by SCD in childhood (3). Previous work has examined coinheritance of other genetic factors (e.g., α thalassemia), laboratory findings (e.g., baseline fetal hemoglobin), and social-environmental factors (e.g., temperature, physical activity, access to healthcare) as drivers of disparate clinical outcomes, though characterization of the phenotypic diversity within SCD remains incomplete (4–8).

Exposure to air pollutants is well characterized as a driver of disparate health outcomes in other health settings, with clinical effects ranging from worse cardiovascular disease, higher rates of asthma and other lung disease, and poor birth outcomes (9–12). While there are many distinct pollutants, the most well known to cause adverse health effects in humans is fine particulate matter (PM_2.5_), particles with a diameter of 2.5 μm or less. From a pathophysiologic perspective, PM_2.5_ causes both local damage via direct lung injury and systemic harm via induction of an inflammatory response, oxidative stress, and endothelial damage (13–19). Notably, these same pathophysiologic pathways (i.e., inflammation, endothelial damage, oxidative stress, and lung injury) are drivers of the severe morbidity and early mortality observed in people with SCD (3, 20, 21). Furthermore, in the American context, SCD is concentrated in the Black community, and predominately Black neighborhoods are exposed to disproportionately high levels of PM_2.5_ (22).

Despite the overlapping pathophysiologic pathways and sociodemographic factors that suggest PM_2.5_ may be especially harmful to people with SCD, research directly examining the link between PM_2.5_ and SCD morbidity is relatively scant (23). Published studies have examined the effect of daily ambient (outdoor) air pollution on numbers of emergency department (ED) visits among groups of patients with SCD, with most finding positive associations between ED visits and higher daily ambient air pollution concentrations (24–28). These studies represent important first steps, though they have key limitations. While the data used in these studies were population-wide, these studies did not include individual-level variables such as SCD genotype, medication use, other laboratory or stroke-risk imaging outcomes, or sociodemographic details. From a pollution viewpoint, these studies did not examine the effects of long-term air pollution exposures on SCD-associated outcomes. Because SCD is a chronic disease, it is plausible that long-term PM_2.5_ exposure, as opposed to daily fluctuations, has more substantial clinical effects. This study aimed to address these critical gaps by providing a comprehensive, individual-level examination of the association between long-term air pollution exposure and health outcomes in children with SCD. We hypothesized that annual PM_2.5_ concentrations at a child’s home would be significantly associated with higher number of ED visits, hospital days, markers of inflammation, and likelihood of abnormal stroke-risk imaging among children with severe SCD (HbSS/HbSβ0).

## Results

There were 1,089 children with severe SCD (HbSS/HbSβ0) who were seen for a clinical encounter at CHOA from January 1, 2010, through December 31, 2019, who fit inclusion/exclusion criteria (Supplemental Figure 1; supplemental material available online with this article; https://doi.org/10.1172/jci.insight.190648DS1). The cohort had average length of follow-up of 5.1 years (range 2–10 years), for a total of 5,531 patient-years. Table 1 shows the descriptive statistics for the sample of interest.

The primary exposure of interest was annual PM_2.5_ values at the child’s home. The variability of this exposure across time and location, which is exploited in our statistical model, is demonstrated in Supplemental Figure 2 and shows that children experienced a range of exposure contrasts (0 to >4 μg/m^3^) over their course of follow-up. The main driver of this PM_2.5_ variability was the overall decrease in PM_2.5_ levels over the study time frame (Supplemental Figure 3), following national trends of air quality improving during the 2010–2019 period. In addition, 216 individuals (19.8% of the cohort of interest) changed addresses during the follow up period, which was an additional driver of observed PM_2.5_ variability. Regarding the primary outcomes of interest, annual hospital days and number of ED visits (Supplemental Figure 4) were right skewed.

**Figure 1:**
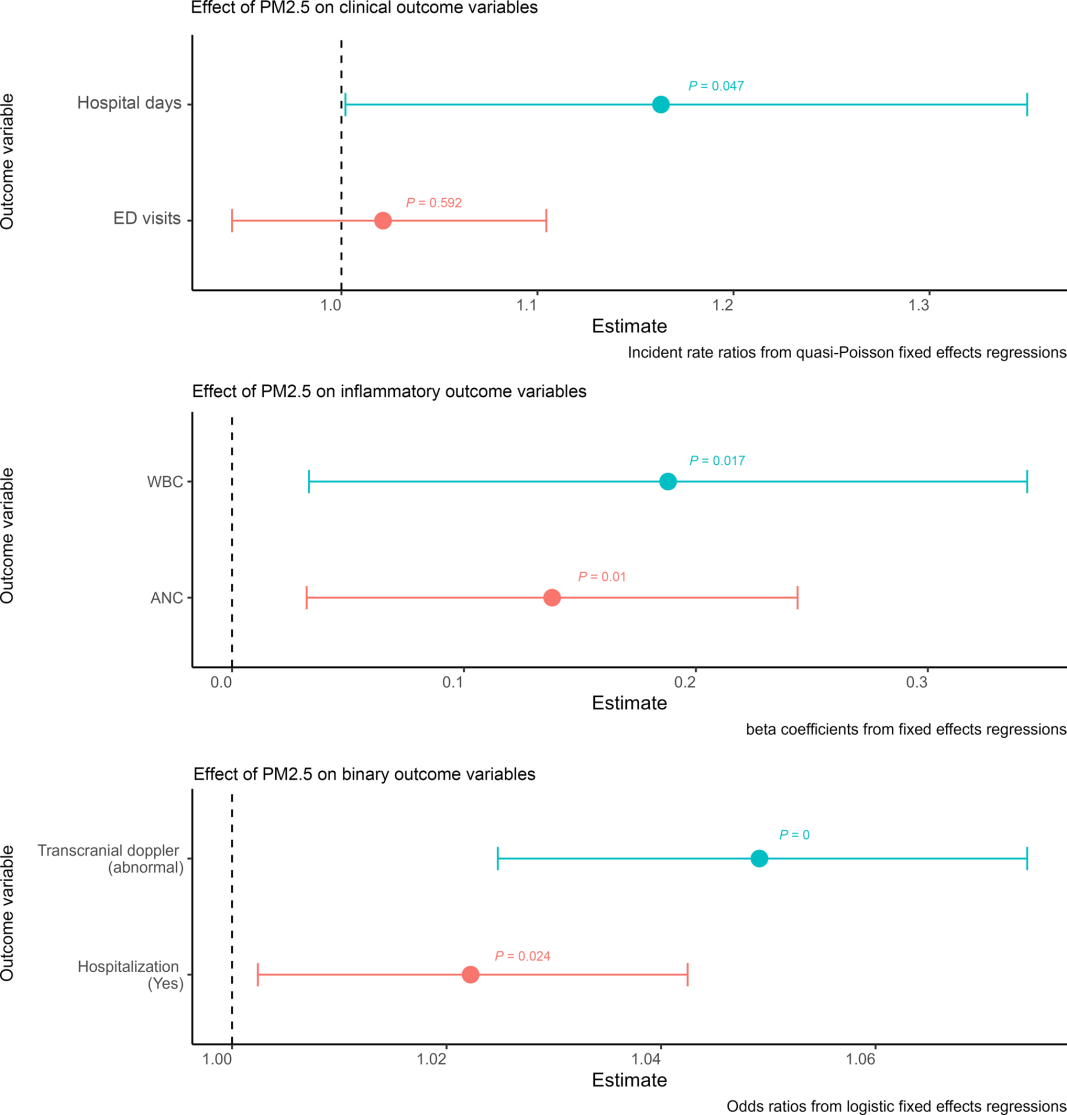
Results of primary models, demonstrating the significant effect of PM_2.5_ on hospital days, inflammatory markers, and abnormal stroke risk screening. This figure shows the main results from the primary models. Each estimate and 95% CI shown comes from a separate model, whose dependent (outcome) variable is labeled on the *y* axis. All models include individual fixed effects and adjust for hydroxyurea use, insurance, census tract social vulnerability index (SVI), distance from hospital, age, and yearly average minimum daily temperature. Clinical outcomes were calculated using quasi-Poisson multivariable models and included 1,089 individuals across 5,531 patient-years. Inflammatory outcomes were calculated using linear (Gaussian) multivariable models and included 1,065 individuals across 4,569 patient-years. Binary outcomes were calculated using logistic multivariable models and included 1,089 individuals across 5,531 patient-years for hospitalizations (yes/no) and 820 individuals across 2,284 patient-years for transcranial Doppler.

Associations of annual PM_2.5_ and the clinical, inflammation-related, and binary outcome variables are presented in Figure 1. After accounting for individual fixed-effects and the covariates/confounders of interest, the following were significantly associated with higher annual PM_2.5_ levels at the individual’s home: number of hospital days per year (incident rate ratio [IRR] = 1.16, *P* = 0.047), likelihood of having a hospitalization in a given year (odds ratio [OR] = 1.02, *P* = 0.024), likelihood of an abnormal stroke-risk screen (transcranial Doppler [TCD]) (OR = 1.05, *P* < 0.001), higher WBC (β = 0.19, *P* = 0.017), and higher absolute neutrophil count (ANC) (β = 0.14, *P* = 0.01). Number of ED visits per year was not significantly associated with annual PM_2.5_ values (IRR = 1.02, *P* = 0.592).

Importantly, as a check of our model, we found that hydroxyurea use decreased WBC and ANC and increased hemoglobin, similar to previously published results on the effect of hydroxyurea on laboratory outcomes (Supplemental Figure 5) (29). Additionally, we performed several sensitivity analyses, including substituting maximum or average for minimum temperature, substituting per capita income for the social vulnerability index (SVI), and including age as a categorical rather than continuous variable; our results were robust to these sensitivity analyses (Supplemental Table 1).

**Figure 2:**
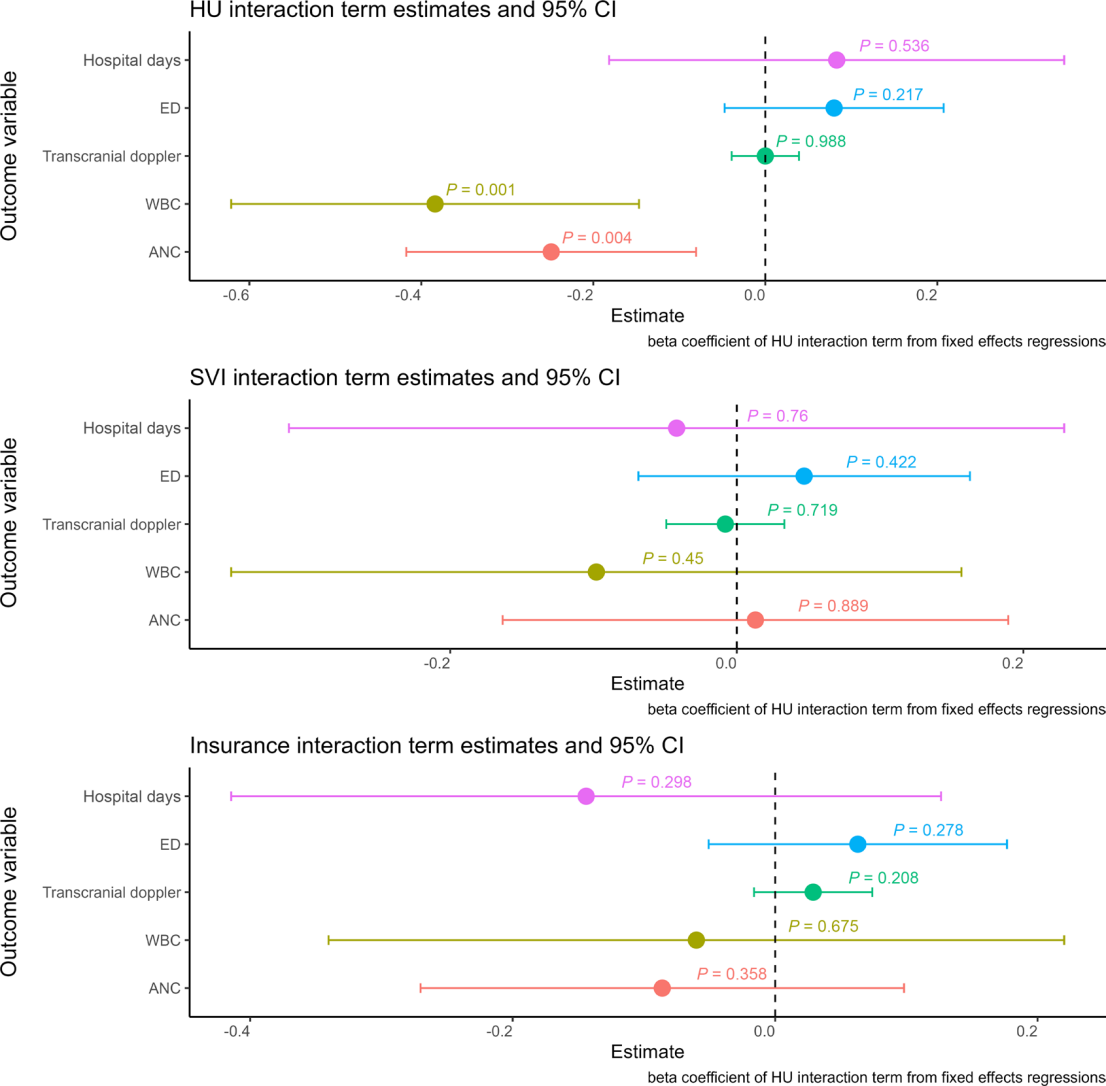
Results of interaction term models, illustrating effect modification by hydroxyurea (HU) use, SVI, and insurance status. This figure shows the main results of the models with interaction terms. Each estimate and 95% CI shown comes from a separate model, whose dependent (outcome) variable is labeled on the *y* axis. All models include individual fixed effects and are adjusted for HU use, insurance, census tract social vulnerability index (SVI), distance from hospital, age, and yearly minimum temperature and contain the interaction terms as shown above. For interaction terms, SVI was dichotomized to above versus below 50th percentile, and insurance was dichotomized to private/commercial versus other. Note that HU use mitigated the inflammatory effects of PM_2.5_ but did not change the effects of PM_2.5_ on clinical outcomes or stroke risk screening. Clinical outcomes were calculated using quasi-Poisson multivariable models, and included 1,089 individuals across 5,531 patient-years. Inflammatory outcomes were calculated using linear (Gaussian) multivariable models and included 1,065 individuals across 4,569 patient-years. Binary outcomes were calculated using logistic multivariable models and included 1,089 individuals across 5,531 patient-years for hospitalizations (yes/no) and 820 individuals across 2,284 patient-years for transcranial Doppler.

In separate models, we included interaction terms to assess whether the estimated effect of PM_2.5_ on outcomes was lower among patients (a) using hydroxyurea, (b) living in census tracts with low SVI, or (c) with private insurance (as proxies for high socioeconomic status). As demonstrated in Figure 2, interaction terms of hydroxyurea use and PM_2.5_ were significantly negative for inflammatory markers (WBC and ANC), consistent with our hypothesis suggesting that hydroxyurea use may mitigate the inflammatory effect of PM_2.5_. However, interaction terms of hydroxyurea use and PM_2.5_ were not significant for the clinical or stroke-risk imaging (transcranial Doppler) outcomes. The interaction term estimates for SVI and insurance status with PM_2.5_ were also statistically insignificant, suggesting no considerable modification of PM_2.5_ effects by these factors.

**Figure 3:**
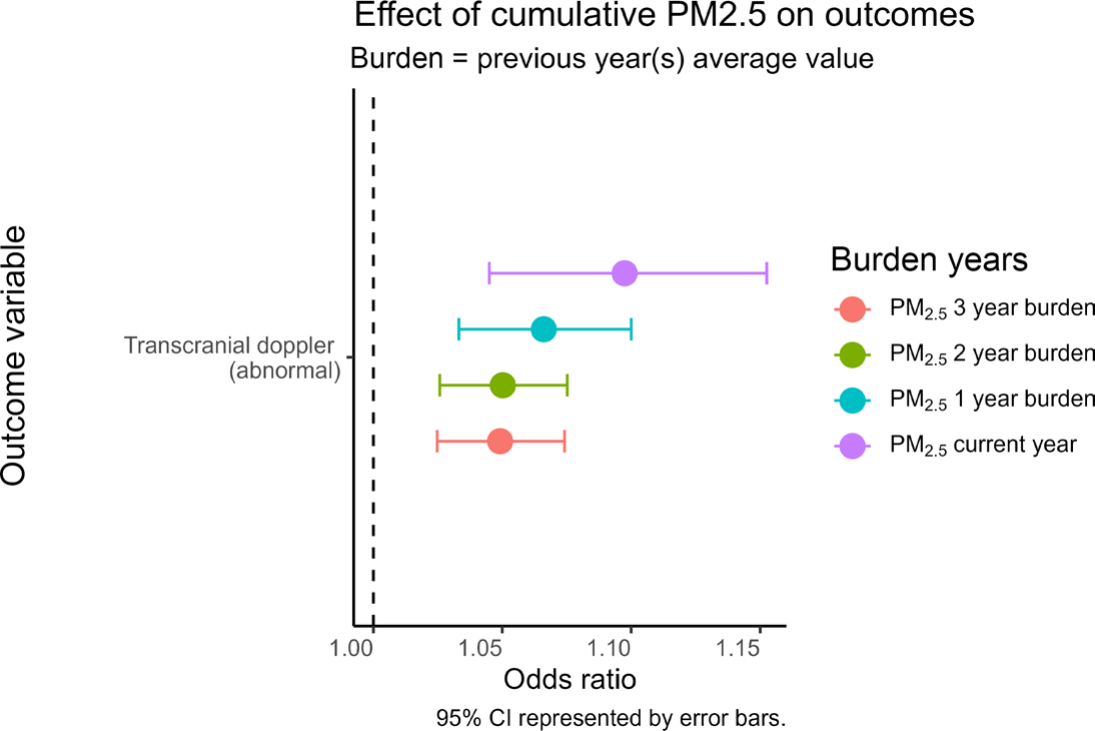
Effect of prior PM_2.5_ burden on likelihood of abnormal stroke risk screening (transcranial Doppler), demonstrating more significant effects with increasing PM_2.5_ exposure time frame. This figure shows the effect of PM_2.5_ burden, which is defined as the average PM_2.5_ values at the home address across 3 years prior, 2 years prior, and 1 year prior to the year of interest, on the likelihood of an abnormal transcranial Doppler ultrasound. Note that transcranial Doppler ultrasound is the standard means of screening for stroke risk in children with SCD and is recommended for every child between 2 and 16 years of age with HbSS/HbSβ0. Abnormal screening, defined as a velocity of 200 cm/s or higher, is associated with significantly increased risk of stroke and has important treatment implications. Transcranial Doppler outcomes were calculated using logistic multivariable models and 820 individuals across 2,284 patient-years for current and 1-year burden, 682 patients across 1,750 patient-years for 2-year burden, and 587 patients across 1,398 patient-years for 3-year burden.

As opposed to current-year annual PM_2.5_ concentrations, prior PM_2.5_ exposures did not have significant effects on hospital days, WBC, or ANC (Supplemental Figure 6). However, prior PM_2.5_ exposures were significantly associated with likelihood of abnormal stroke-risk screening, with longer-exposure time frames demonstrating increasing association (Figure 3).

**Figure 4:**
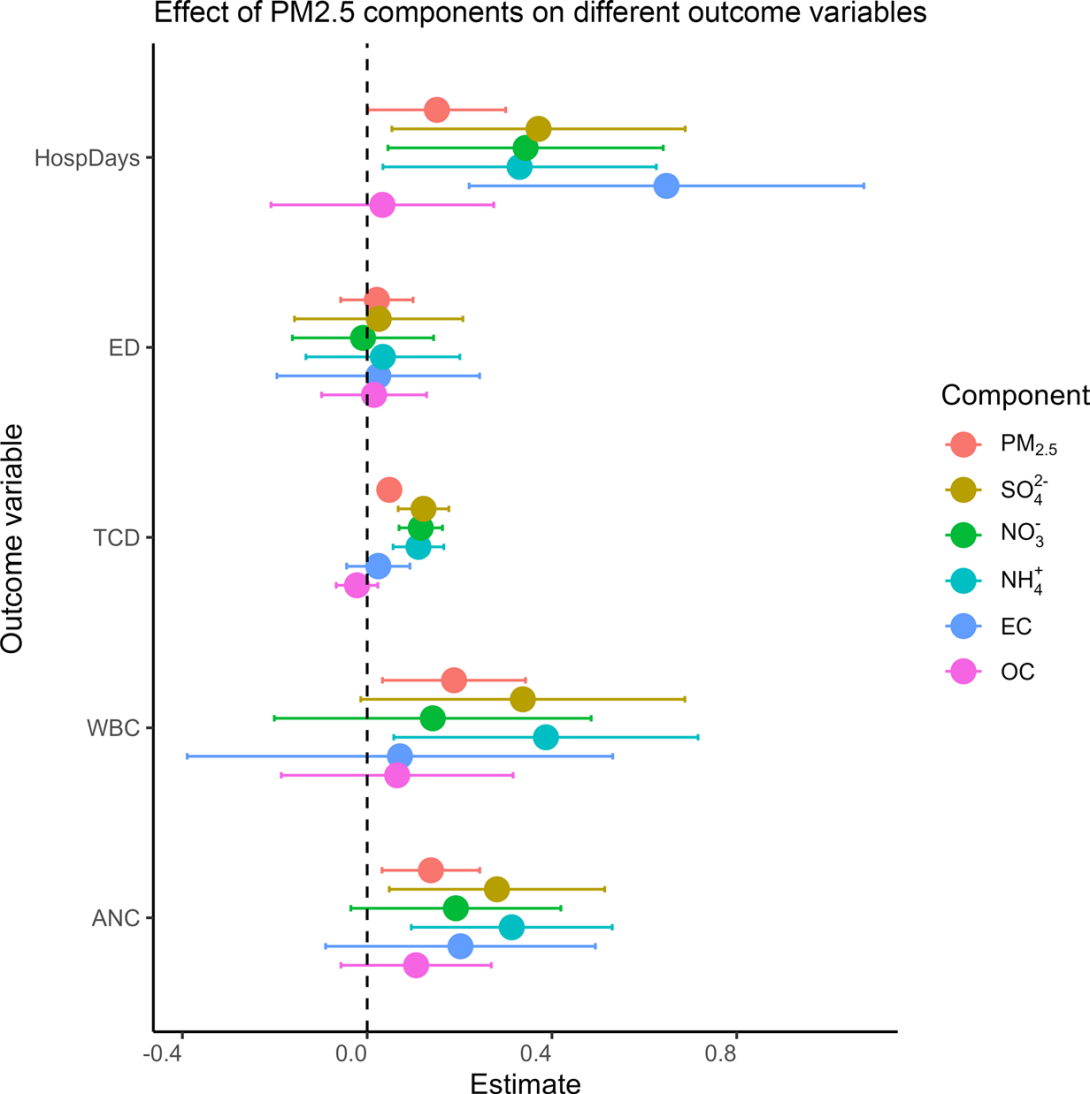
Effect of PM_2.5_ components on different outcomes. Each β coefficient estimate and 95% CI shown on the *x* axis represents a unique fixed effects model, where the outcome of interest is shown on the *y* axis, and the exposure of interest is a different PM_2.5_ component, including elemental (black) carbon (EC), organic carbon (OC), ammonium (NH_4_^+^), sulfate (SO_4_), and nitrate (NO_3_). For comparison, PM_2.5_ components have been standardized (mean centered and divided by their SD). Clinical outcomes were calculated using quasi-Poisson multivariable models and included 1,089 individuals across 5,531 patient-years. Inflammatory outcomes were calculated using linear (Gaussian) multivariable models and included 1,065 individuals across 4,569 patient-years. Binary outcomes were calculated using logistic multivariable models and included 1,089 individuals across 5,531 patient-years for hospitalizations (yes/no) and 820 individuals across 2,284 patient-years for transcranial Doppler.

Different components of PM_2.5_ have been associated with varying health effects in other settings (30). To determine whether this variability also occurs among children with SCD, we conducted a secondary analysis estimating the effects of individual PM_2.5_ components. Figure 4 illustrates that, although the estimated effects varied among the components, SO_4_ and NH_4_ were the most strongly associated with inflammatory markers (WBC and ANC). Organic carbon (OC) demonstrated no statistically significant effect across any of the examined health outcomes. The concentrations of PM_2.5_, SO_4_, NH_4_, and NO_3_ were spatially correlated; elemental carbon (EC) and OC showed less correlation (Supplemental Figure 7).

Lastly, the results from our supplementary analysis, which includes patients with HbSC disease, can be found in Supplemental Table 2. Briefly, the associations between PM_2.5_ and clinical outcomes were generally weaker in the HbSC group compared with those with HbSS/HbSβ0. While increased PM_2.5_ was significantly associated with more hospital days and higher WBC and ANC counts in the HbSS group, these associations were attenuated and largely nonsignificant in HbSC, consistent with the lower levels of inflammation and hemolysis typically observed in HbSC disease.

## Discussion

In this retrospective, longitudinal analysis of 1,089 children with severe SCD encompassing 5,531 individual-years, high overall levels of ambient PM_2.5_ exposure were observed, with the mean annual PM_2.5_ exposure (9.8 μg/m^3^) at the child’s home above the current national ambient air quality standard of 9.0 μg/m^3^ (31). In the fixed effects analyses, annual PM_2.5_ concentrations were significantly associated with worse clinical outcomes (e.g., hospital days per year, likelihood of abnormal stroke-risk screening via transcranial Doppler) and higher annual values for inflammatory markers (WBC and ANC). Notably, hydroxyurea use acted as an effect modifier for inflammatory markers, though this effect modification was not observed for clinical and stroke-risk screening outcomes. Observed associations were largely similar for major PM_2.5_ components, including secondary PM_2.5_, SO_4_, and NH_4_, suggesting these to be important drivers of the overall PM_2.5_ effect and potential areas for targeted environmental health policy.

Consistent with our primary hypotheses, higher annual PM_2.5_ concentrations were associated with worse outcomes. In other words, our results demonstrate that, for an individual, residing in an area with higher annual PM_2.5_ values was associated with worse outcomes for that individual. These findings are both statistically and clinically significant, with a 1-unit increase of PM_2.5_ associated with an incidence rate ratio of 1.163, or 16.3% increase in expected hospital days, holding other factors constant. To put this in perspective, in February 2024, the Environmental Protection Agency reduced the annual PM_2.5_ National Ambient Air Quality Standard from 12 to 9 μg/m^3^ (31); in the context of our fixed effects Poisson regression model, a reduction of PM_2.5_ of this magnitude would be associated with an expected decrease in the incidence rate of hospital days of 36.5% for a child whose baseline PM_2.5_ exposure is 12 μg/m^3^. Notably, the World Health Organization has more stringent standards, recommending annual PM_2.5_ exposure of 5 μg/m^3^; given our results, we would expect to see even further improvements in health outcomes in children with SCD if the United States adopted these recommendations.

Beyond solely confirming previous work that documents harms of PM_2.5_ on clinical outcomes, our findings extend the literature in several key areas. Most prior literature, especially with regard to SCD, has focused on the effect of short-term (e.g., daily) fluctuations in air pollutant levels, with De et al. as a notable exception (25–27, 32). In contrast, the exposures of interest in this study were annual PM_2.5_ levels. Annual PM_2.5_ levels are a critical exposure metric because they reflect the sustained environmental conditions that individuals face, which is particularly relevant for chronic diseases like SCD where long-term environmental factors may influence disease progression and management. Furthermore, it is annual PM_2.5_ levels that have been the subject of recent policy changes in the United States (31). Understanding the distinct health effects of PM_2.5_ components can lead to more precise public health interventions and policies, enhancing protection for sensitive groups like children with SCD. Since the Environmental Protection Agency is mandated to provide standards that protect the health of all populations, including vulnerable populations (e.g., children with SCD), it is imperative that rigorous data on the effects of long-term pollutant exposure are well documented. This long-term perspective can reveal cumulative health effects that short-term–exposure assessments might miss, providing a more comprehensive understanding of how persistent air pollution exposure affects health outcomes over time.

One key way in which the long-term perspective is highlighted in this study is through our findings related to transcranial Doppler screening, which assesses stroke risk in children with SCD. Our results demonstrate that chronic exposure to elevated PM_2.5_ levels is significantly associated with an increased likelihood of abnormal TCD screening, indicating heightened stroke risk. This correlation between air pollution exposure and abnormal stroke-risk screening aligns with previously reported correlations between PM_10_ and elevated internal carotid artery blood velocity in children with SCD (33). Notably, beyond SCD, prior research has demonstrated strong associations between PM_2.5_ exposure and increased stroke risk in the general population (34, 35). Furthermore, endothelial injury has been described as a potential mechanism, providing a strong biologic basis for this association (36). Stroke is one of the most severe and life-threatening manifestations of SCD in pediatric populations, underscoring the critical nature of this finding. Moreover, we observed a dose-response relationship, with longer durations of exposure to higher PM_2.5_ levels being progressively associated with a greater likelihood of abnormal TCD results (Figure 3). Importantly, hydroxyurea use did not mitigate the effect of PM_2.5_ on the likelihood of abnormal stroke-risk screening. The observed cumulative dose-response relationship suggests that PM_2.5_ exposure may cause cumulative and clinically relevant endothelial damage in children with SCD and suggests that hydroxyurea might not be sufficient to prevent this adverse outcome. This relationship further emphasizes the cumulative effect of sustained PM_2.5_ on stroke risk in this vulnerable population. Clinically, these abnormal TCD findings have substantial management implications, as children with abnormal stroke-risk screening are recommended more aggressive interventions such as chronic transfusion therapy or, in some cases, bone marrow transplantation to prevent stroke occurrence.

Notably, ED visits were not correlated with annual PM_2.5_ concentrations. One potential explanation for the differential outcomes observed between ED visits and hospital days lies in the nature of these metrics. ED visits typically signify acute exacerbations, while hospital days may be indicative of more severe underlying disease. Previous research has demonstrated a correlation between ED visits and acute increases in daily air pollution levels for children with SCD (26, 27), suggesting that acute rises in pollution levels are likely to trigger immediate health issues, leading to an increase in ED visits. Conversely, chronic exposure to elevated PM_2.5_ levels appears to exacerbate the severity of the underlying disease, resulting in more frequent and/or longer hospital stays. This hypothesis aligns with our data and highlights the distinct effect of acute versus chronic exposure to air pollution on health outcomes in children with SCD.

Another unique strength of this study lies in its longitudinal analysis of a cohort of children with SCD. These data and modeling strategy allowed for tracking of individuals over time. The fixed effects multivariable model takes advantage of this panel data and controls for time-invariant confounders, such as baseline SCD severity and underlying genetics. This methodology strengthens our ability to make causal inferences about the effect of long-term PM_2.5_ exposure on health outcomes in children with SCD. Additionally, by including hydroxyurea use as an interaction term, our study investigated whether this medication was an effect modifier. We found that hydroxyurea significantly reduced the effect of PM_2.5_ on 2 markers of inflammation, WBC and ANC, although it did not alter the effects on clinical outcomes, including hospital days, ED visits, and stroke-risk screening. One potential explanation for this discrepancy is that clinical outcomes represent more distal endpoints, influenced by a broader range of biological and behavioral factors beyond inflammation. In contrast, WBC and ANC are more direct measures of systemic inflammation, which hydroxyurea is known to attenuate. Additionally, variability in healthcare utilization and other unmeasured factors may have contributed to the lack of observed effect modification on these clinical endpoints. These considerations underscore the complexity of the relationship between air pollution, inflammation, and clinical disease in SCD, while suggesting a potentially new avenue for mitigating air pollution-related harms, beyond traditional avoidance strategies.

There are several limitations that warrant mention. First, our focus on CHOA’s patient data, though comprehensive, may not fully capture the experiences of children with SCD outside the Atlanta (Georgia, USA) metropolitan area. Second, our outcomes, hospital days, and ED visits do not fully capture the experience or severity of SCD, and WBC and ANC are imperfect markers of inflammation. Future studies that more precisely document the effect of air pollution on SCD severity and inflammation are needed. Third, while our fixed effects model controlled for unobserved individual time-invariant factors, they did not account for any unobserved time-varying factors, such as indoor pollution or unrecorded fluctuations in individual health behaviors or socioeconomic status; thus, there is a possibility for residual confounding if such factors are correlated with ambient PM_2.5_ levels. Furthermore, although we controlled for socioeconomic status using a census tract-level SVI and insurance status, these measures may not fully capture the multidimensional aspects of socioeconomic status. Thus, routine collection of additional socioeconomic indicators at the individual level, such as household income, parental education, and employment status, both inside and outside of clinical trials, could assist in reducing residual confounding and in better characterizing the relationship between socioeconomic factors, environmental exposures, and health outcomes in SCD. Fourth, air pollution exposures were assessed based on home address only; we were not able to characterize children’s overall exposures to ambient PM_2.5_ that account for time-activity patterns such as time spent at school and other locations. Lastly, our findings are derived from a single healthcare system encompassing multiple hospitals. While this setting ensures relatively uniform care practices and treatment protocols, it also limits generalizability. Differences in environmental exposures, sociodemographic factors, and healthcare access across other regions and/or countries may influence the relationship between air pollution and SCD outcomes. Future studies incorporating multiinstitutional or national datasets would help validate these findings in broader and more diverse populations.

In conclusion, in this longitudinal study of 1,089 children with SCD, we identified significant associations between annual PM_2.5_ exposure and adverse clinical and laboratory outcomes, underscoring the importance of addressing air quality in vulnerable populations. Notably, we found that long-term PM_2.5_ exposure was associated with more hospital days and an increased likelihood of abnormal transcranial Doppler screenings, with stroke risk rising progressively with more cumulative PM_2.5_ exposure. The study’s innovative approach, particularly the fixed effects methodology and examination of hydroxyurea as an effect modifier, opens new avenues for research and intervention beyond traditional pollution avoidance strategies. Future studies are needed that examine the effect of long-term air pollution exposure and hydroxyurea use on more precise inflammatory markers and respiratory-specific outcomes.

## Methods

### Sex as a biological variable.

Both male and female patients were included in the study population. We did not consider sex as a relevant biological variable in this analysis.

### Data sources.

Patient-level data were abstracted from the electronic medical records in an ongoing, longitudinal cohort of children with SCD at CHOA, a network of hospitals, urgent care, and outpatient clinics constituting the largest pediatric hospital system and subspecialty care provider in Georgia (USA). Briefly, this cohort includes all children with SCD (as verified by hemoglobin analysis), who have at least 1 clinical encounter at CHOA. Importantly, CHOA accounts for ~95% of pediatric SCD hospitalizations in the Atlanta (Georgia, USA) metropolitan area; therefore, the data included represent a nearly complete population-based sample of children with SCD in Atlanta (37). Patient information abstracted included clinical, laboratory, and sociodemographic (including home address at each encounter) data.

Our analysis included pediatric patients (age < 18 years at time of encounter), from January 1, 2010, through December 31, 2019, with the first and last time points being the first and last clinical encounters that occurred within this period, up to the child’s eighteenth birthday. We included only patients with HbSS/HbSβ0, the most common and severe forms of SCD. Given that key endpoints in our study included stroke screening and hydroxyurea use, both of which are not routinely performed or commonly prescribed in HbSC disease, we restricted our primary analysis to patients with HbSS/HbSβ0. However, to explore potential differences by genotype, we conducted a supplementary analysis including patients with HbSC, with results presented in the Supplement.

We censored children once their home address was listed as either unknown or > 30 miles from the nearest CHOA facility. We also censored children if they underwent bone marrow transplant and/or gene therapy, given different risk and utilization profiles. Lastly, children were excluded if they had insufficient clinical data in our system, including < 3 clinical visits (inpatient + ED + outpatient) in total, to limit the study population to children who would likely use CHOA as their primary source of inpatient and outpatient care (Supplemental Figure 1).

For socioeconomic status, we integrated the Centers for Disease Control and Prevention’s SVI, matching each child with the census-tract level SVI (38). The SVI is an index that incorporates various census-tract level indicators, including socioeconomic status, household composition, minority status, and housing type, allowing for a nuanced assessment of social vulnerability.

Air pollution data were acquired from the NASA Socioeconomic Data and Applications Center (SEDAC), which provides publicly available data on key pollutants. We specifically utilized the annual mean PM_2.5_ and PM_2.5_ component datasets, which combine remote sensing (satellite) and ground-level monitoring data into a machine learning algorithm to provide annual, high resolution (1 × 1 km for PM_2.5_, 50 × 50 m for PM_2.5_ components) pollutant concentrations (39). Annual PM_2.5_ values (and PM_2.5_ components) were matched to each child using the child’s geocoded home address for the given year. Annual weather data came from National Climatic Data Center (40).

### Measures.

The primary exposures of interest were annual PM_2.5_ concentrations, assigned for each child based on values for the 1 × 1 km grid cell in which their home was located each year. If a child changed addresses during the year, we calculated the annual PM_2.5_ concentration as the average across the grid cells in which they resided, weighted by days at each address. As a secondary analysis, we estimated the effect of long-term, lagged PM_2.5_ exposures on the outcomes of interest by averaging PM_2.5_ values at the home across 3 years prior, 2 years prior, and 1 year prior. For example, the 3-year average value for a 5-year-old child was calculated as the average of annual PM_2.5_ values at the child’s home for age-years 2, 3, and 4, with the outcomes of interest (e.g., annual hospital days, average WBC value) occurring at 5 years of age.

As secondary exposure analyses, we estimated the effect of PM_2.5_ components on the outcomes of interest. We focused on 5 major components: EC, often referred to as black carbon and a marker for diesel exhaust; OC, which includes a vast array of organic compounds arising from combustion processes; ammonium (NH_4_^+^), which typically originates from agricultural sources and traffic; sulfate (SO_4_), which is mainly derived from the burning of fossil fuels; and nitrate (NO_3_), also a common byproduct of fossil fuel combustion and agricultural activities (12). Exposure assignment methodology was consistent for PM_2.5_ and its components, utilizing annual concentrations based on the child’s home address.

The primary outcomes of interests were measures of SCD clinical severity, including number of inpatient hospital days and ED visits per year of age (e.g., from 2.00 to 2.99 years of age). To mitigate the influence of extreme outliers due to prolonged hospitalizations in few children, statistical outliers for hospital days were Winsorized, meaning that values above the 95th percentile were replaced with the value at the 95th percentile. Given the known impact of PM_2.5_ on the endothelium and inflammation, secondary outcome variables of interest included abnormal stroke screening by transcranial Doppler (abnormal versus conditional/normal per year), and markers of inflammation including WBC and ANC. Laboratory values were annual, averaged for each patient across values each year taken at baseline (i.e., during an outpatient well visit).

### Statistics.

Univariate and bivariate analyses were conducted to assess the distributions and associations of our primary exposures and outcomes. Next, a fixed effects model was implemented to investigate the relationship between air pollution exposure and SCD clinical severity within our panel dataset. The estimating equation is shown below,

*Y_it_* = α*_i_* + β*_1_* pollution*_it_* + β*_2_* hydroxyurea*_it_* + β*_3_* age*_i_* + β*_4_* insurance*_it_* +β*_5_* distance*_it_* + β*_6_* SVI*_it_* + β*_7_* temperature*_t_* + ε*_it_*

where *Y* is the outcome of interest for individual *i* at age-year *t* (e.g., 2 years of age, 3 years of age), α*_i_* is the individual fixed effect, and β*_1_* is the primary coefficient of interest. The individual fixed effect α*_i_* represents a unique intercept for each individual, accounting for their inherent baseline level of the outcome variable before considering other covariates. Covariates of interest were chosen a priori based on potential associations with air pollution exposure and/or SCD severity, and included: age (continuous variable, chosen as continuous due to worsening SCD severity with age and because modeling age as continuous and linear would help account for trends across time in the fixed-effects model), insurance (private versus Medicaid versus uninsured), distance to nearest hospital (continuous), SVI (continuous, higher number represents higher vulnerability), and annual average daily minimum temperature. Hydroxyurea use, defined as reporting hydroxyurea use for more than half of all clinical visits for a given age year, was included as a covariate in the primary models (without effect modification). In separate models, hydroxyurea use was included as an interaction term with annual pollution exposure, testing the hypothesis that the antiinflammatory properties of hydroxyurea would mitigate the harms from PM_2.5_ exposure. Proxies of socioeconomic status, including insurance use and census-tract SVI, were also measured as effect modifiers, to examine whether families with higher socioeconomic status may be able to better mitigate the harmful effects of air pollution (e.g., through better home air filtration systems). For interaction terms, SVI was dichotomized to above versus below the 50th percentile, and insurance was dichotomized to private/commercial versus other. The fixed-effects models assign a unique fixed effect to each person, effectively controlling for unobservable and time-invariant individual characteristics that could confound the relationship between air pollution exposure and clinical and laboratory outcomes in SCD (41). Via this model, we were able to focus on variations in air pollution exposure levels and their effect on SCD clinical severity across different time points for the same individuals. This approach minimizes the bias in our estimates that could arise from omitted variables specific to each person, such as genetic factors or long-term health conditions, by comparing the same individual under different conditions of exposure. Consequently, this model enhances the reliability of our findings by using the within-individual changes over time to reveal the causal relationship between air pollution and SCD severity, while holding all unobserved, individual-specific factors that do not vary over time as constant. Given the precise nature of our exposure data — namely, time-varying, annual pollutant concentrations matched to each individual’s home address and adjusted for any address changes during the study period — this model is particularly appropriate for examining the specific effect of air pollution on health outcomes among this cohort of children with SCD. Count outcome variables were analyzed using a quasi-Poisson regression, continuous variables were assessed through linear regression, and dichotomous outcome variables were examined with logistic regression models (42). Given repeated measurements, standard errors were clustered at the individual level to account for within-individual correlation. Children with missing outcome or primary exposure data were excluded from the relevant analysis. As a check on the validity of the fixed-effects model, we reviewed the estimated effect of hydroxyurea use on WBC, ANC, and hemoglobin, whose effects are well documented in the literature (29, 43). All analyses were performed in R v4.3.3.

### Study approval.

This study was approved by the CHOA IRB.

### Data availability.

We are committed to promoting transparency and reproducibility in research. To facilitate this, we will make the code used in this manuscript available to the scientific community (https://github.com/pegeorge/Publication_Coding/). Due to IRB restrictions and the necessity to protect Protected Health Information, the identifiable data underlying this study cannot be publicly shared. However, aggregated data supporting the findings are available upon reasonable request to qualified researchers in accordance with our data-sharing policies. Values for all data points in graphs are reported in the Supporting Data Values file.

## Author contributions

PEG served as the primary author, overseeing the research project, and coordinating the manuscript preparation. GK assisted with data collection and conducted data analysis. JL was responsible for drafting and editing the manuscript. DHH provided editing support and offered feedback on statistical analysis and study design. BK contributed to the conception of the study and participated in manuscript editing. WAL oversaw the final draft, provided supervision, and assisted with editing. SE aided in study design, provided supervision, and helped edit the manuscript.

## Supplementary Material

Supplemental data

ICMJE disclosure forms

Supporting data values

## Figures and Tables

**Table 1:**
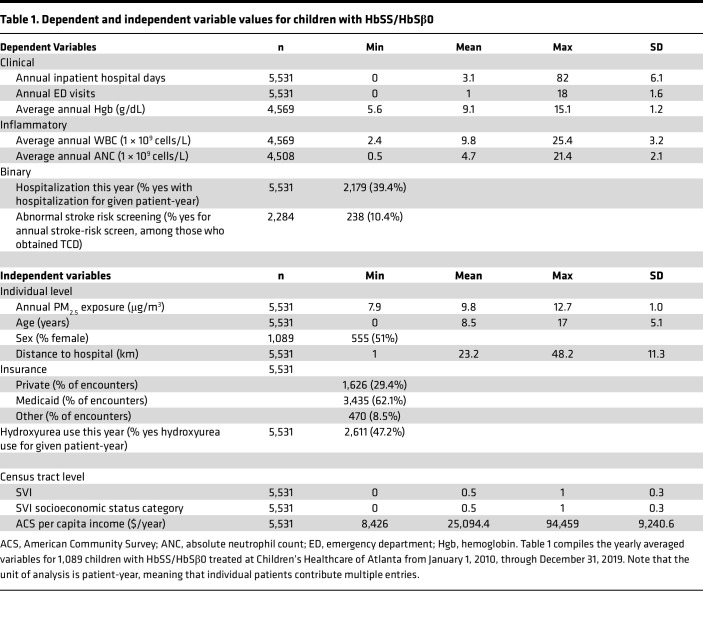
Dependent and independent variable values for children with HbSS/HbSβ0

## Version 1 06/09/2025

Electronic publication
