# Supplementary material for: Annual PM2.5 exposure and clinical, laboratory, and stroke-risk outcomes in pediatric sickle cell disease
Source: JCI Insight. 2025 Jun 9;10(11):e190648. doi: 10.1172/jci.insight.190648 (PMC12220963; doi:10.1172/jci.insight.190648)
Supplement: Supplemental data [file jciinsight-10-190648-s082.pdf]

Supplemental material

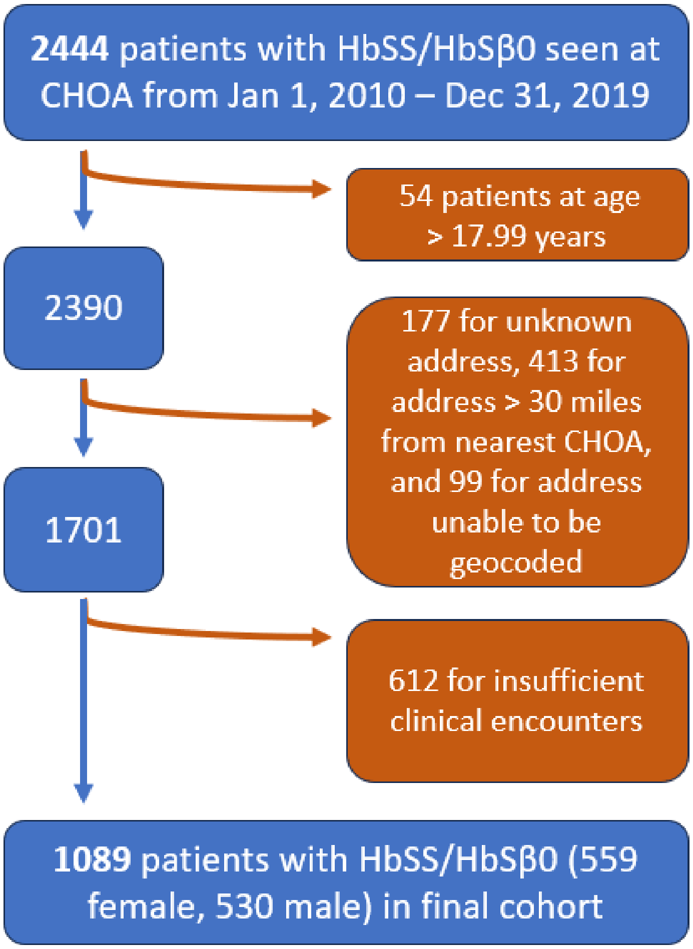

Supplemental Figure 1: Patient Inclusion/Exclusion Flow Chart. CHOA: Children's Healthcare of Atlanta. Insufficient clinical encounters: < 2 years of follow up, or < 3 clinical visits in total (excluding encounters for bone marrow transplant).

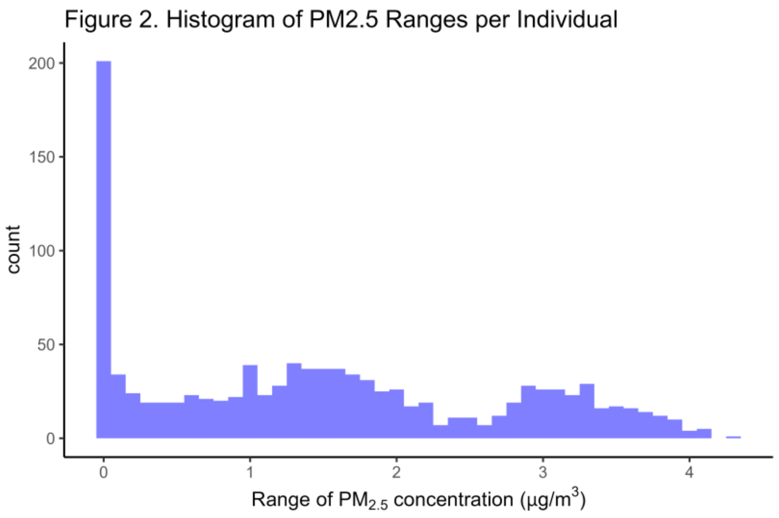

Supplemental Figure 2: Individual $PM_{2.5}$ variation in cohort. This figure shows the $PM_{2.5}$ range for each individual in the cohort over their course of follow up in the study, where range is defined as the maximum minus the minimum annual $PM_{2.5}$ concentration (µg/m^3) at the individual's home address.

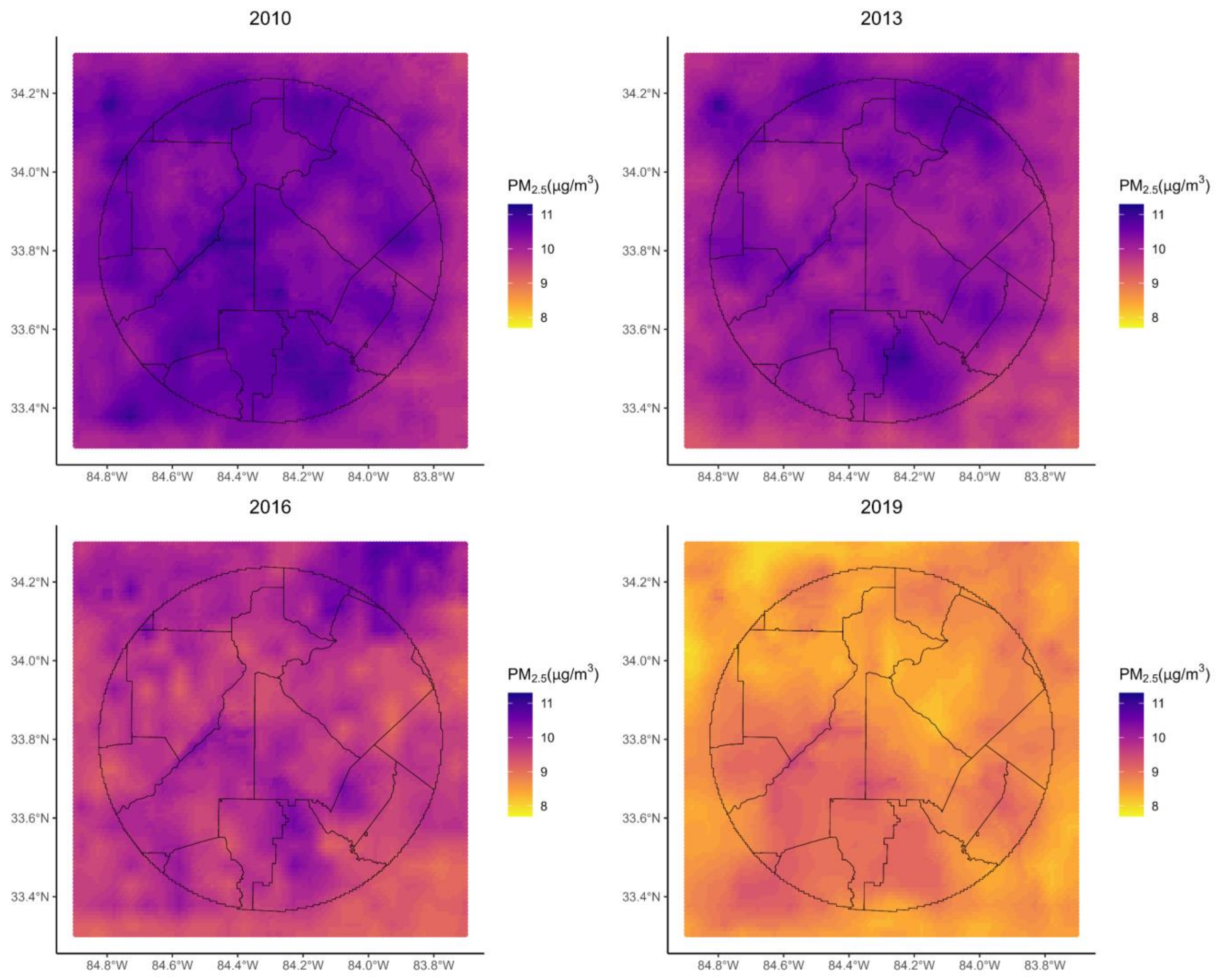

Supplemental Figure 3, Change in $PM_{2.5}$ across Atlanta, GA, from 2010 to 2019. This figure shows the change in annual $PM_{2.5}$ concentration across Atlanta, GA. The color scale is the same for each panel. The black lines show the county outlines within Atlanta. The outer black circle represents 30 miles from the midpoint of the 3 Children's Healthcare of Atlanta (CHOA) hospitals. Annual $PM_{2.5}$ data was abstracted from the NASA Socioeconomic Data and Applications Center (SEDAC) Annual $PM_{2.5}$ Concentrations Database.

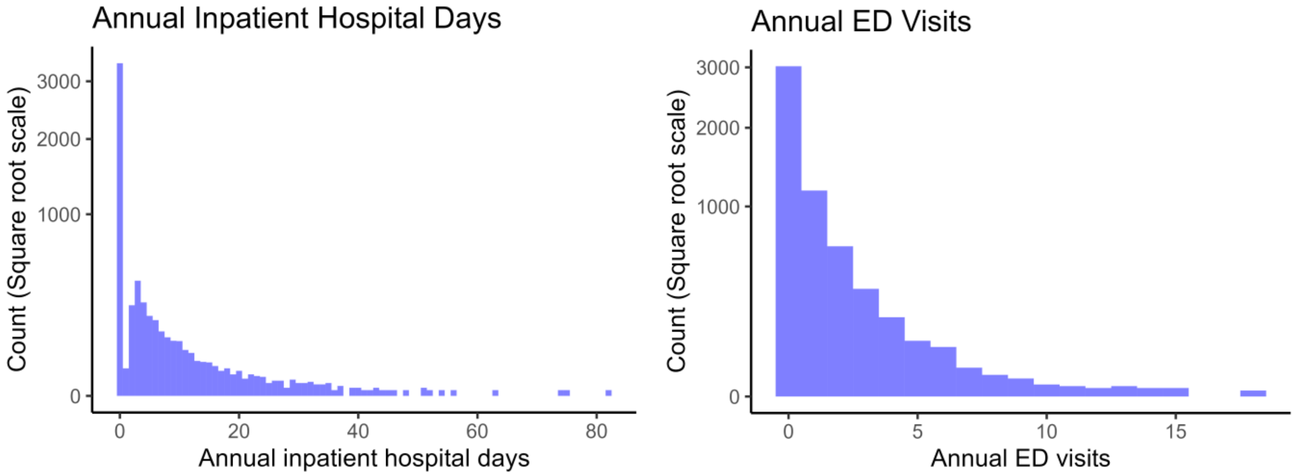

Supplemental Figure 4, Distribution of Inpatient and Emergency Department (ED) Visits. This figure displays the primary clinical outcomes of interest, annual inpatient days and annual number of ED visits. Note that or inpatient days, there appear to be two distributions – one for patients who never have a hospitalization, and another for patients who have at least one hospitalization. This bimodal distribution is not observed for annual ED visits.

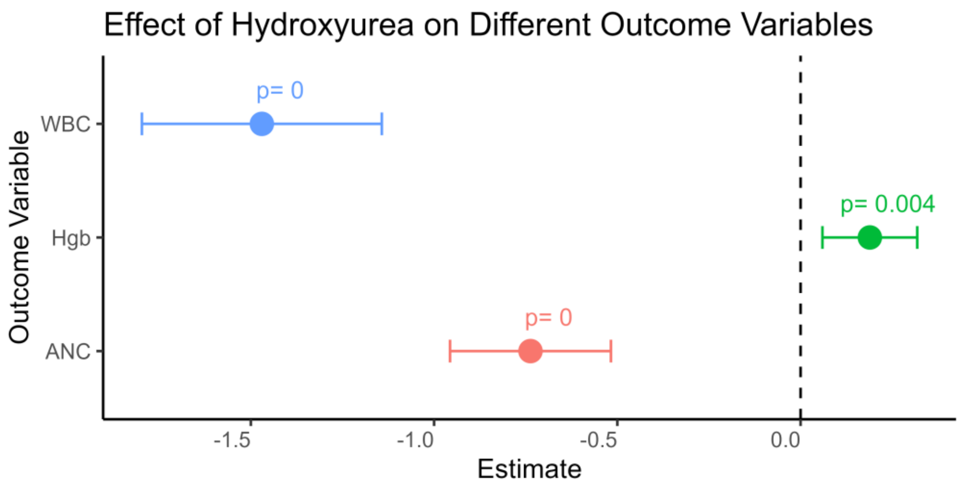

Supplemental Figure 5, Effect of hydroxyurea on laboratory outcomes. These results serve as a test on the fixed effects model, as it is well-described that hydroxyurea use reduces white blood cell count (WBC) and absolute neutrophil count (ANC), and increases hemoglobin (Hgb). The magnitude and direction of the values observed in this figure are consistent with published results.

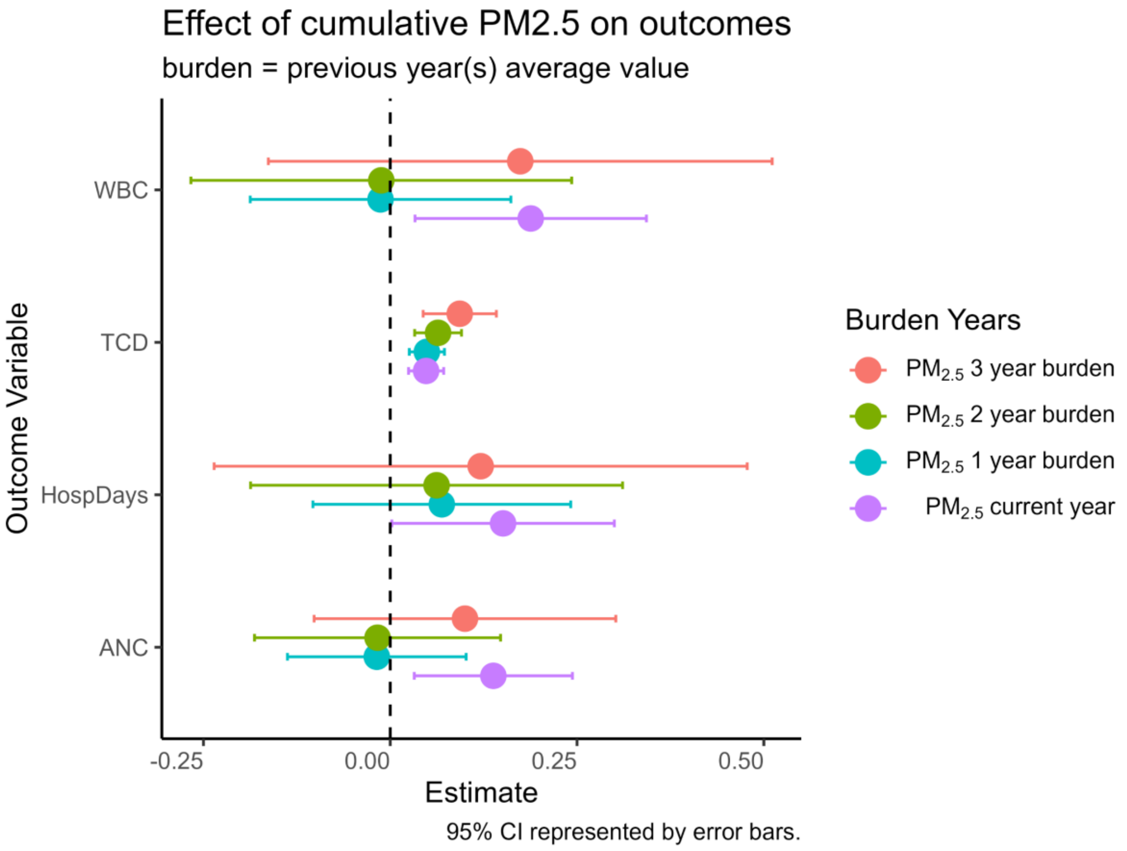

Supplemental Figure 6, Effect of prior $PM_{2.5}$ burden on different outcomes. This figure shows the impact of $PM_{2.5}$ burden, which is defined as the average $PM_{2.5}$ values at the home address across 3 years prior, 2 years prior, and 1 year prior to the year of interest. For example, the 3-year burden value for a 5-year-old patient would be the average of annual $PM_{2.5}$ values at the patient's home address for 2, 3, and 4 years of age, with the outcomes of interest (e.g., annual hospital days, average WBC value) occurring at 5 years of age.

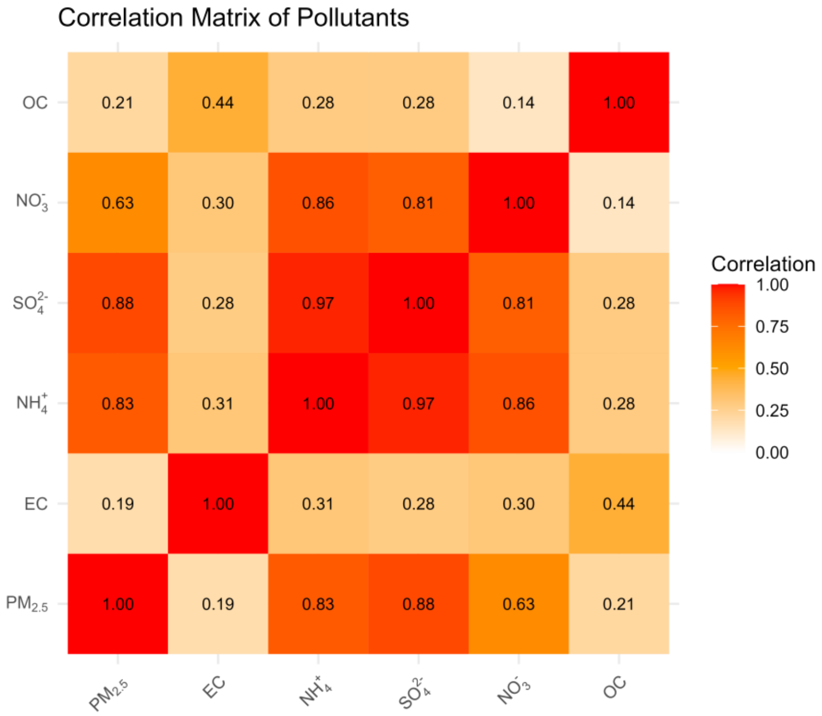

Supplemental Figure 7: Correlation matrix of PM2.5 and components. As expected, all pollutants are positively correlated.

Supplemental Table 1: Comparison of results from sensitivity analyses.

Model	Outcome	$PM_{2.5}$ Estimate	Standard Error	p-value
Primary Model	Hospital days	0.151	0.076	0.047
Max Temp Model	Hospital days	0.166	0.077	0.031
Avg Temp Model	Hospital days	0.168	0.075	0.026
Per Cap Income Model	Hospital days	0.136	0.076	0.075
Age Cat Model	Hospital days	0.16	0.074	0.032
Primary Model	ED visits	0.021	0.04	0.592
Max Temp Model	ED visits	0.025	0.04	0.525
Avg Temp Model	ED visits	0.023	0.038	0.537
Per Cap Income Model	ED visits	0.018	0.04	0.658
Age Cat Model	ED visits	0.014	0.04	0.719
Primary Model	WBC	0.188	0.079	0.017
Max Temp Model	WBC	0.186	0.077	0.016
Avg Temp Model	WBC	0.188	0.077	0.015
Per Cap Income Model	WBC	0.174	0.079	0.028
Age Cat Model	WBC	0.147	0.078	0.059
Primary Model	ANC	0.138	0.054	0.01
Max Temp Model	ANC	0.132	0.053	0.013
Avg Temp Model	ANC	0.136	0.053	0.01
Per Cap Income Model	ANC	0.131	0.054	0.016
Age Cat Model	ANC	0.097	0.049	0.046
Primary Model	Abnormal TCD	0.048	0.012	<0.001
Max Temp Model	Abnormal TCD	0.042	0.012	<0.001
Avg Temp Model	Abnormal TCD	0.036	0.012	0.002
Per Cap Income Model	Abnormal TCD	0.048	0.012	<0.001
Age Cat Model	Abnormal TCD	0.048	0.012	<0.001
Primary Model	Hospitalization (Yes)	0.022	0.01	0.024
Max Temp Model	Hospitalization (Yes)	0.024	0.01	0.013
Avg Temp Model	Hospitalization (Yes)	0.025	0.01	0.012
Per Cap Income Model	Hospitalization (Yes)	0.02	0.01	0.044
Age Cat Model	Hospitalization (Yes)	0.024	0.01	0.012

This table shows the $PM_{2.5}$ estimate results on the primary model and sensitivity analyses, which substitute maximum temperature and average temperature for minimum temperature, and substitutes area deprivation index and census tract per capita income for social vulnerability index, and includes age as a categorical instead of continuous variable.

ANC: absolute neutrophil count; Cat: categorical; ED: emergency department; TCD: transcranial doppler; WBC: white blood cell.

Results of HbSC analysis

In total, there were 558 patients with HbSC (268 (48%) female), with an average of 6.2 years in the dataframe.

Supplemental Table 2: Comparison of HbSS and HbSC Outcomes

	HbSS/Sbeta0				HbSC			
Outcome	Estimate	Standard error	p-value	number of observations	Estimate	Standard error	p-value	number of observations
Hospital days (count)	1.163	0.076	0.047	5531	1.088	0.103	0.417	3049
ED days	1.021	0.04	0.592	5531	0.974	0.057	0.645	3049
WBC	0.188	0.079	0.017	4569	0.087	0.084	0.3	2126
ANC	0.138	0.054	0.01	4508	0.179	0.084	0.033	1891
Hospital days (binary)	1.022	0.01	0.024	5531	1.016	0.017	0.358	3049

This table presents the estimated associations between ambient PM2.5 levels and various clinical outcomes in two distinct patient groups, those with sickle cell anemia (SCA) and those with the HbSC genotype. For each outcome, the table displays the point estimate (with exponentiated values reported as incidence rate ratios for the quasipoisson and binomial models—interpreted as odds ratios in the case of the binary outcome—and the raw coefficient for the Gaussian models), the corresponding robust standard error (clustered at the patient level), the p-value, and the number of observations used in each analysis.
